# Thermal Volume Reduction Surgery for Surgical Treatment of Pulmonary Bullae: A Single-Center Treatment Experience of 276 Cases Accompany With Primary Lung Cancer

**DOI:** 10.3389/fsurg.2021.672688

**Published:** 2021-05-04

**Authors:** Tianjian Lu, Weiping Lu

**Affiliations:** ^1^Department of Thoracic Surgery, West China Hospital, Sichuan University, Chengdu, China; ^2^Department of Thoracic Surgery, Jilin Cancer Hospital, Changchun, China; ^3^Changchun Tumor Hospital, Changchun, China

**Keywords:** lung volume reduction surgery, thermal ablation, lung, pulmonary bullae, electrocautery

## Abstract

**Objective:** Lung volume reduction surgery (LVRS) has been regarded as an effective surgical procedure for severe emphysema (including pulmonary bullae). However, there still remain controversial that its applications limited that only patients with a specific clinical situation may benefit from LVRS, and so did other non-surgical treatments. The current study aims to introduce some initial experience of new technique for treating pulmonary bullae, including using thermal surgical instruments to reduce enlargement of lung tissue in a specific group that diagnosed with lung cancer accompany with pulmonary bullae.

**Methods:** This retrospective study included 276 patients undergoing emphysema reducing surgery between 2010 and 2020. All procedure were performed by thermal volume reduction surgery of using thermal surgical instruments to reduce pulmonary bullae.

**Results:** The average time required for operating single pulmonary bullae was <10 min. Median operative time was 106 min (range 85 to 191 min). No intraoperative air leak, massive blood loss, or other severe complications occurred. The estimated blood loss for TVRS was about 40 ml (range 15 to 120 ml). Postoperative complications included atelectasis (*n* = 8), pulmonary infection (*n* = 17), bleeding (*n* = 5), delayed air leak (*n* = 7) among the cohort. The postoperative lung function at 1-year post surgery in TVRS group recovered faster with a better recovery that achieving an FEV1 of 1.95 ± 0.46 L, TLC of 6.36 ± 0.79 L, RV of 3.56 ± 0.81 L, PO_2_ of 60 ± 8 mmHg, PCO_2_ of 37 ± 6 mmHg, and 6 MWD (6-min walk distant) of 305 ± 22 m. The 1-year QOL score was elevated comparing with preoperative period.

**Conclusion:** This single-center study reported a new thermal-based surgical approach to treat pulmonary bullae by reducing abnormally enlarged lung tissue in specific patients diagnosed with lung cancer accompany with pulmonary bullae.

**Graphical Abstract:**
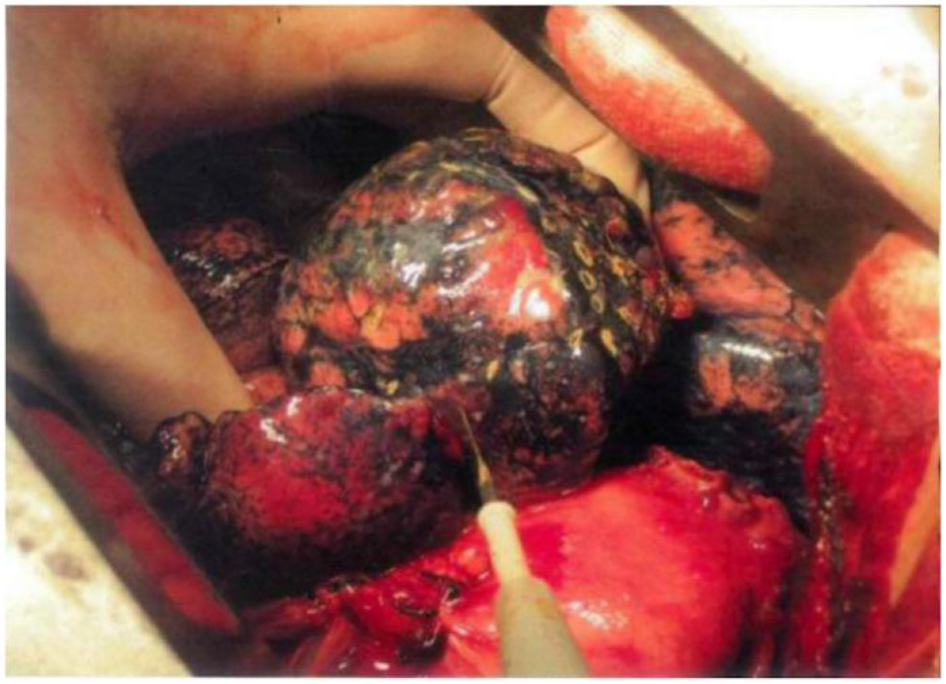
Use electrocautery to undergo the burning procedure to the surface of pulmonary bullae.

## Highlights

- This report introduced a lung volume reduction procedure by electrocautery and other thermal ablation procedure.
- Thermal Volume Reduction Surgery (TVRS) had a satisfactory outcome for lung volume reduction in lung cancer patients.
- Thermal Volume Reduction Surgery (TVRS) had a optimizing surgical outcome for treating advanced emphysema.

## Introduction

Emphysema is a pathological clinical manifestation of long-term damage to lung function and irreversible dilatation of the terminal bronchioles and alveoli (1). Enlargement of the airspace structure is the main pathological change associated with emphysema, and severe cases may develop into pulmonary bullae, which eventually lead to acute rupture of the lungs (2). Surgery is an effective therapeutic method for severe emphysema, aiming to remove dysfunctional lung tissues, reduce the ventilation–perfusion mismatch, and improve diaphragm function (3).

Lung volume reduction surgery (LVRS) is a surgical procedure commonly used for removing dysfunctional lung tissues (4). LVRS is fundamentally the same as subpulmonary lobectomy. However, LVRS has a high risk of adverse complications, including prolonged air leaks, pneumonia and acute inflammation, which may increase short-term mortality (5). In recent years, implantation of endobronchial Valves and Coils has been approved as a novel treatment for emphysema (6, 7). In 1996, McKenna et al. (8) conducted a randomized trial in which diffuse emphysema patients were treated by laser bullectomy. The results showed a negative outcome from the use of a Nd:YAG contact laser. Thermal vapor ablation has also recently been applied for emphysema (9–11). Gompelmann et al. (12) reviewed the clinical application of bronchoscopic thermal vapor ablation, suggesting the potential value of thermal procedures in the treatment of emphysema or pulmonary bullae.

Here, we report an improvement in the use of an electric cautery procedure, namely, thermal volume reduction surgery (TVRS), as an alternative to lung tissue dissection for the treatment of emphysema. In principle, this procedure may both ensure the effectiveness of surgical treatment and reduce early-stage morbidity and mortality **by** reducing lung tissue resection. Notably, we assessed the therapeutic efficacy of TVRS in primary lung cancer patients with pulmonary bullae located in different lobes on the same side to reduce the unnecessary dissection of benign lung tissue.

## Patients and Methods

### Patients

**Figure 1:**
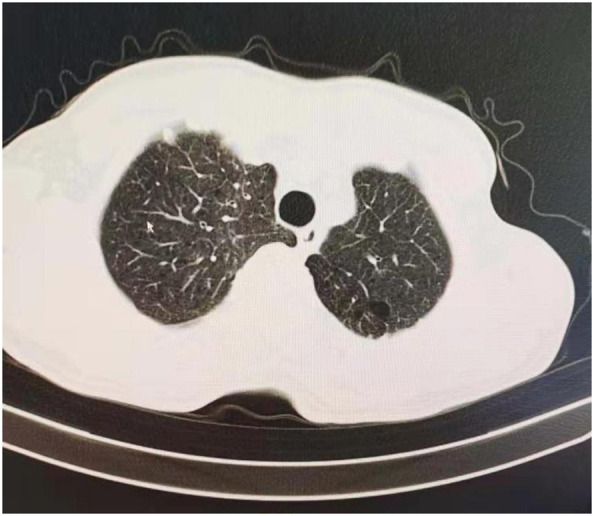
CT screening of included patient, this case had pulmonary bullae located at left upper lobe.

This study was approved by the Institutional Review Board of Jilin Cancer Hospital (No. 201412-028). Written informed consent was obtained preoperatively from lung cancer patients recruited in our hospital from May 2010 to May 2020. The average age at surgery was 63 years (51–76 years). All patients were diagnosed with primary lung cancer with advanced emphysema or pulmonary bullae on the same side. The inclusion criteria were as follows: (1) Lung cancer and enlargement of lung tissues were preoperatively confirmed by low-dose computed tomography (LDCT), and the pulmonary bullae were near the visceral pleura (Figure 1), and the diameter of pulmonary bullae was not <1.5 cm according to the results of the imaging examination. (2) The pulmonary bullae were located on the same side as the primary lung tumor but not in the lobe invaded by the tumor. (3) Preoperative lung function was strong enough that the patient could tolerate radical resection of the lung tumor. The basic characteristics and perioperative outcomes of included patients were collected to analyze. The postoperative outcomes and quality of life (QOL) was focused on the recovery of pulmonary function in 30 days and 1 years. The QOL were evaluated by St-George Respiratory Questionnaire (SGRQ) (13) improved version. The scoring criteria were summarized by 4 parts (Activities of daily living, Social activities, Depression and Anxiety). In this scoring system, the score is inversely proportional to the quality of life. All the included data in our study was from these 276 patients.

### Operation Procedure

The surgical incision was made based on the surgical choice of lung tumor resection, which was not altered by TVRS. Both Video-assisted surgery (VATS) and open surgery are appropriate for the TVRS procedure. The types of surgery used for tumor resection included wedge resection, segmentectomy, and lobectomy.

#### Procedure Selection and Preparation for Thoracic Surgery

**Figure 2:**
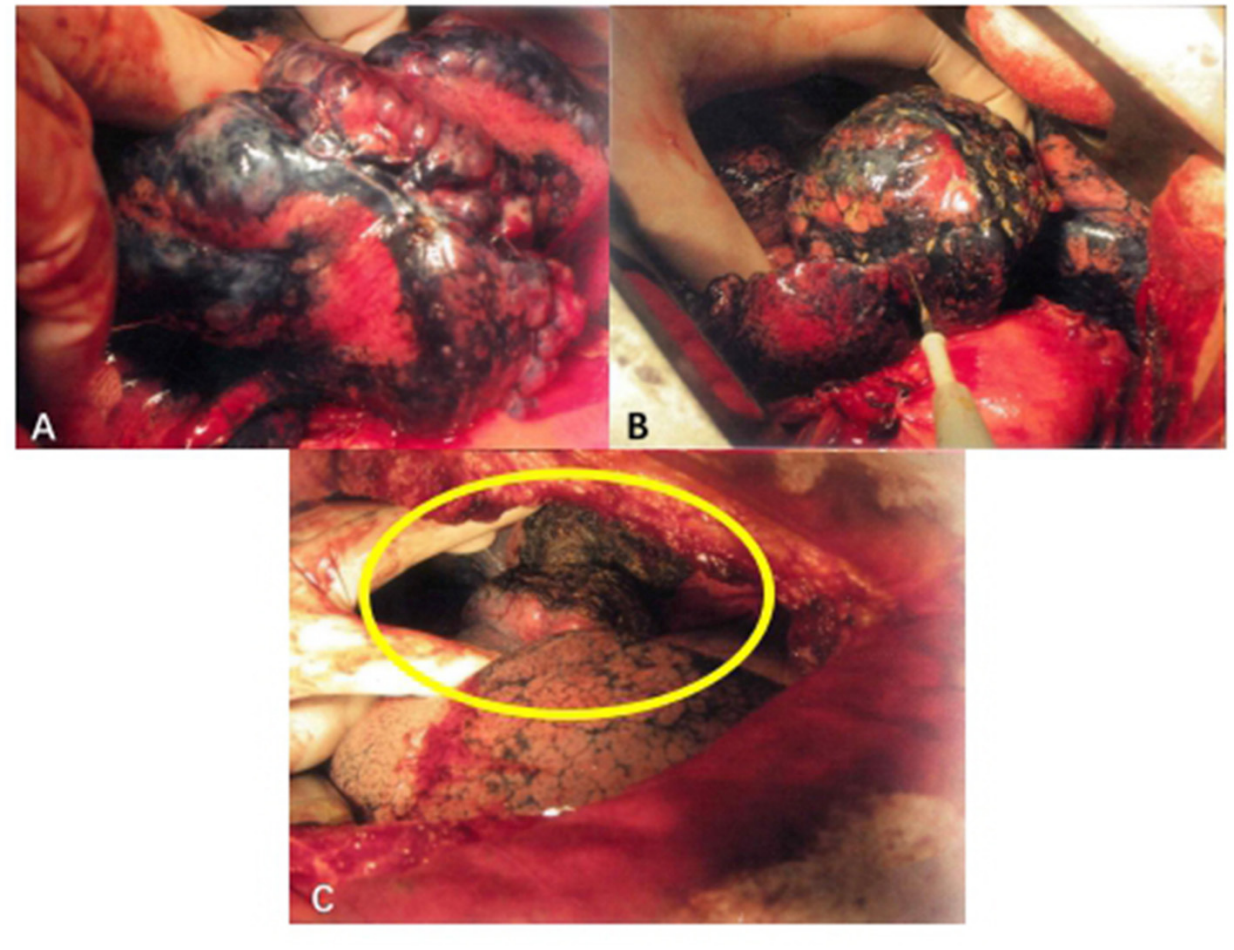
Surgical procedure of TVRS. **(A)** Pulmonary bullae of lung under operative field. **(B)** Use electrocautery to undergo the burning procedure to the surface of pulmonary bullae. **(C)** After the procedure, the abnormally enlarged lung volume will decrease to normal shape (yellow circle).

LDCT was preoperatively performed (the threshold was set to −950 EU), and clinical information including the number, location, and pleural adhesion of the bullae and the degree of destruction [evaluated by the emphysema index (EI), which indicates the percentage of low-attenuation area in the total pulmonary volume], was collected. Patients with emphysema may have an abnormal increase in lung volume, and the pressure of local pulmonary bullae increases unevenly, leading to difficulty in the local operation. An example of the surgical view of pulmonary bullae for open surgery is shown in Figure 2A, and the whole procedure is shown in Supplementary Video 1. The hyperinflation of pulmonary bullae should be confirmed by both the imaging examination results and the surgical view. Because this procedure does not require special preparation of surgical instruments, surgeons may evaluate intraoperatively whether this procedure can be performed.

In the open surgical procedure, the target lung was normally ventilated to expose the emphysema more clearly and to ensure the safety of patients with poor lung function. Airway pressure was continuously monitored during the surgical procedure. Double-lumen endotracheal tubes were used during anesthesia. In the VATS procedure, low-flow ventilation could be performed on the target lung to ensure sufficient operation space.

#### Surgical Technique for Emphysema

Electric cautery was applied to burn the surface of the emphysema site (Figure 2B). The extension of the cautery area was determined by the preoperative CT scanning result and intraoperative observation of the volume of pulmonary bullae. The whole surface of the bulla was burned, and the cauterized area extended no more than 0.5 cm beyond the boundary of the bulla. The whole cautery procedure was deemed complete once the bullae shrank to the point that they no longer compressed the surrounding lung tissue. Under this procedure, the wall of the emphysema lesion shrank, the volume of the emphysema lesion decreased, and the abnormally enlarged lung eventually shrank to a normal volume (Figure 2C). The following parameters were set: electric cautery = 30 W (output power); distance between cautery burning points = 1–2 cm on the same pulmonary bulla. After volume reduction, the compression of the healthy lung tissue was relieved, and the remaining lung tissue recovered its normal function. The local temperature was over 100°C, and the burning operation was performed carefully to prevent delayed air leakage caused by destruction of the lung tissues through cautery. Since the thickness of the lung tissue on the surface of pulmonary bullae was relatively low and the bulge was obvious, direct use of an electric knife may lead to rupture of lung tissues, resulting in air leakage. If operating conditions permitted, 2 or 3 layers of gauze soaked in normal saline (NS) were applied over the operating surface to cool the tip of the cautery iron, thus avoiding damage to the lung tissues. Then, the burning procedure continued in this manner, aiming to ensure that the lung tissues covered by gauze were evenly heated and that the local temperature was not excessively high. The airway pressure should be stable during the burning procedure. If an air leak occurred, the airway pressure decreased, at which time the lung tissues needed to be repaired immediately to prevent prolonged air leakage.

#### Postoperative Protection of Lung Tissues

The thoracic cavity and lung surface were flushed with normal saline (NS). The density and toughness of burnt lung tissues were enhanced, and no special material was needed to protect the surgical surface. Meanwhile, due to the increased toughness of the lung tissue, the rate of local rupture of the lung tissue decreased when the airway pressure was increased by coughing.

### Statistical Analysis

All the variables were evaluated by *t*-test. Measurement data that follow a normal distribution are expressed as $\bar{x}$ ± s. *P* < 0.05 was considered statistically significant. SPSS Statics software ver. 25.0 (IBM Corporation, NY, USA) was applied to perform all statistical analyses.

## Results

From May 2010 to May 2020, a total of 276 eligible patients were chosen from 675 lung cancer patients who underwent surgery in Jilin Cancer Hospital. Their baseline information is shown in Table 1. All recruited patients were diagnosed with primary lung cancer with advanced emphysema, which was clinically manifested as simple or multiple pulmonary bullae located in different lobes from the tumor site. The average time required to operate on single pulmonary bullae was <10 min, and the median operative time was 106 min (85–191 min). No intraoperative air leakage, massive blood loss or other severe complications occurred. The estimated blood loss during TVRS was ~40 ml (15–120 ml). Postoperative complications included atelectasis (*n* = 8), pulmonary infection (*n* = 17), bleeding (*n* = 5), and delayed air leakage (*n* = 7) (Table 2). At 1 year postoperatively, the TVRS group had recovered its lung function more effectively than the other group, achieving an FEV1 of 1.95 ± 0.46 L, TLC of 6.36 ± 0.79 L, RV of 3.56 ± 0.81 L, PO_2_ of 60 ± 8 mmHg, PCO_2_ of 37 ± 6 mmHg, and 6 MWD (6-min walk distant) of 305 ± 22 m (Table 3). The 1-year QOL score was elevated comparing with preoperative period (Table 4).

**Table 1:** Pre-operative clinical characteristics of patients included in series.

**Characteristics (*n =* 276)**		**Number (%)**
**Age at diagnosis (years)**		
	Median (IQR)	63 (42–79)
	Mean	61 ± 12
**Gender**		
	Male	165 (59.8)
	Female	111 (40.2)
**Main symptoms**		
	No symptoms	63 (22.8)
	Dyspnea	132 (47.8)
	Cough	35 (12.7)
	Chest pain	30 (10.9)
	Expectoration	16 (5.8)
**History of COPD**		
	No	97 (35.1)
	Yes	179 (64.9)
**Smoking status**		
	Never	74 (26.8)
	Former smoker	108 (39.1)
	Current smoker	94 (34.1)
**Emphysema location**		
	Left upper lobe	73 (26.4)
	Left lower lobe	53 (19.2)
	Right upper lobe	70 (25.4)
	Right middle lobe	35 (12.7)
	Right lower lobe	45 (16.3)

**Table 2:** Intraoperative and postoperative outcomes after TVRS surgery in included series.

**Variables (*n =* 276)**		**Number (%)**
**Surgery type for tumor**		
	Wedge resection	28 (10.1)
	Segmentectomy	54 (19.6)
	Lobectomy	194 (70.3)
**Surgical approach**		
	Open surgery	47 (17.0)
	Video-assisted surgery (VATS)	189 (68.5)
	VATS convert to Open	40 (14.5)
**Operative time (min)**		
	Median (IQR)	106 (85–191)
	Mean	123 ± 27
**Blood loss (ml)**		
	Median (IQR)	40 (15–120)
	Mean	65 ± 25
**Postoperative complications**		
	Atelectasis	8 (2.9)
	Pulmonary infection	17 (6.2)
	Bleeding	5 (1.8)
	Delayed air leak	7 (2.5)

**Table 3:** Preoperative and postoperative lung function in included series.

**Variable**	**Preoperative**	**Postoperative (30d)**	**t**	***P***	**Postoperative (1 year)**	**t**	***P***
FEV1 (L)	2.03 ± 0.52	1.78 ± 0.36	5.442	0.032	1.95 ± 0.46	3.270	0.005
TLC (L)	7.83 ± 0.72	6.15 ± 0.68	2.736	0.006	6.36 ± 0.79	1.985	0.014
RV (L)	5.32 ± 0.76	3.25 ± 0.73	9.512	0.115	3.56 ± 0.81	7.232	0.049
MVV (L/min)	94 ± 20.1	73 ± 15.6	11.157	0.046	82 ± 18.2	1.163	0.014
PO_2_ (mmHg)	53 ± 6	59 ± 9	−1.168	0.021	60 ± 8	−3.490	0.009
PCO_2_ (mmHg)	42 ± 11	39 ± 8	1.751	<0.001	37 ± 6	2.872	<0.001
6 MWD (m)	264 ± 18	198 ± 15	13.304	<0.001	305 ± 22	−6.685	<0.001

**Table 4:** Preoperative and postoperative Quality of life (QOL) score [Based on St-George Respiratory Questionnaire (SGRQ) improved version].

**Variable**	**Preoperative**	**Postoperative (30d)**	**t**	***P***	**Postoperative (1 year)**	**t**	***P***
Activities of daily living	2.73 ± 0.52	2.04 ± 0.46	5.267	<0.001	1.81 ± 0.32	7.734	<0.001
Social activities	2.41 ± 0.36	1.98 ± 0.31	6.890	0.019	1.65 ± 0.18	11.152	<0.001
Depression score	2.37 ± 0.43	2.21 ± 0.37	2.218	<0.001	2.05 ± 0.31	4.782	0.003
Anxiety score	2.10 ± 0.47	1.98 ± 0.38	2.992	0.002	1.93 ± 0.35	2.110	<0.001
QOL score	73.68 ± 7.21	71.24 ± 7.05	0.875	<0.001	61.04 ± 6.36	8.392	<0.001

### Pathological Results

**Figure 3:**
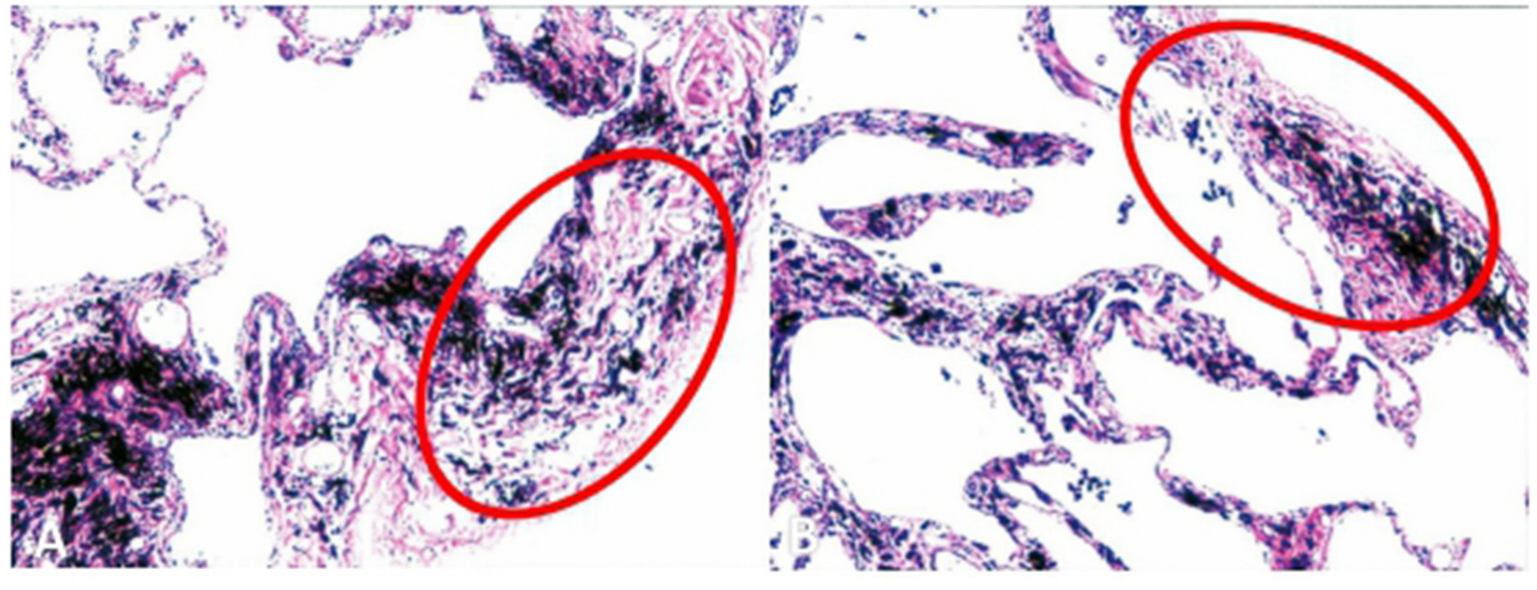
Pleural histological structure after thermal procedure. The visceral pleural of the burned area contracted and thickened obviously, and the whole volume of the alveoli contracted.

Cauterized pulmonary tissue specimens were collected during the surgical procedure to observe the pathological changes that occurred during TVRS. Microscopic observation showed that the visceral pleura of the burned area markedly contracted and thickened, and the total volume of the alveoli contracted. TVRS significantly alleviated pathological changes in pulmonary bullae (Figure 3).

## Discussion

In 1957, Brantigan and Muller (14) first reported a surgical approach known as lung volume reduction surgery, in which the emphysematous part of the lung is resected. The aim is to resect the overexpanding, dysfunctional lung tissues and restore ventilation and perfusion of normal lung tissues, although it has disadvantages such as a high risk of complications and early mortality. At present, the clinical benefits of lung volume reduction surgery remain controversial. The National Emphysema Treatment Trial (NETT) is the largest and most comprehensive multicenter prospective randomized controlled trial (RCT) assessing the LVRS procedure in severe emphysema cases. It has been reported that LVRS is effective in severe emphysema patients and shows acceptable safety in high-risk populations (15). Ginsburg et al. (16) reported a 10-year experience indicating that LVRS is safe and effective for a specific group of patients with emphysema. A recent study from Lim et al. (17) reported that LVRS was more effective than conventional medical treatment in restoring lung function and relieving dyspnea. At present, the nature of a suitable candidate for LVRS is still controversial.

New technologies have gradually developed and expanded the application range of surgical treatment for emphysema. Another novel approach consisting of implantation of endobronchial valves or coils has been recently approved to treat emphysema with intact lobar fissures or absent collateral ventilation (6, 7), although the number of clinical cases is limited. In 2016, Sciurba et al. (18) reported an RCT (RENEW) showing the effectiveness and safety of endobronchial coil treatment for emphysema patients. Slebos et al. (19) identified that CT screening and significant hyperinflation (residual volume≥200% of predicted) are essential for identifying the population that is suitable for endobronchial coil therapy. Other trials (20–22) have also indicated the efficiency of implantation of endobronchial valves. As a result, multiple therapeutic approaches may potentially be effective for advanced emphysema and pulmonary bullae, though the REVOLENS trial (23) indicated that endobrochial coil required a higher short-term cost which may influence the cost-effectiveness. As for thermal therapy, bronchoscopic thermal vapor ablation has been noted for its practical advantages endoscopic lung volume reduction (12), although this procedure has been applied at only a limited group of centers, requiring a special surgical instrument, and it cannot be performed in patients with multiple or confluent pulmonary bullae.

The TVRS operation can be regarded as a surgical procedure intermediate between surgery and interventional therapy, wherein the thoracic cavity is exposed without dissecting any lung tissues. Instead, this procedure is performed to reduce the volume of emphysema lesions using the characteristics of energy-based surgical instruments. We have found that TVRS is feasible in practice for patients with special clinical situations, and the recovery of pulmonary function and the incidence of complications were acceptable. Overall, our cohort was a series of patients with unusual clinical situations. In the current study, all patients were diagnosed with primary lung cancer by imaging examination, and the presence of pulmonary bullae was discovered at the same time. The main pathological characteristics of the recruited patients were ipsilateral primary lung cancer and bullae located in different lobes of the lung. In the case of radical resection of malignant lesions, the expansion of the lung resection range due to accompanied benign lesions may reduce the tolerance to surgery and affect postoperative lung function. Several studies (24–26) also indicated that the postoperative pulmonary function of lung cancer patients decreased in a long-term follow-up period regardless of the surgical procedure. This may lead to a potential risk of postoperative adverse events caused by postoperative pulmonary dysfunction, especially in patients with preoperative chronic pulmonary disease. Therefore, we expected this novel procedure to preserve lung function as much as possible. Emphysema-affected lung tissue may constrict functional lung tissues and limit normal respiratory function. One of the principles of TVRS is that this procedure helps to relieve the compression of lung tissues by emphysema and restore normal lung function, and the postoperative outcome confirmed our prediction. The surgical treatment and prognosis of lung cancer were unaffected by the TVRS procedure, confirming the effectiveness and safety of TVRS. In addition, no special preparations were required for simultaneous surgery on the pulmonary bullae.

Based on these surgical experiences, we can summarize the potential advantages of TVRS for selected patients: (1) Double-lumen endotracheal tubes were used during anesthesia for TVRS, and normal ventilation of the healthy lung was ensured when air leakage occurred in the target lung. (2) Local thermal treatment exerted a bactericidal effect and reduced the risk of postoperative infection. (3) TVRS was able to preserve normal lung tissues to the maximum extent, since there was no need to dissect any lung tissue, and it was simultaneously able to preserve the integrity of lung tissue, especially in our patients who underwent essential lung dissection for lung cancer. (4) The automatic hemostasis procedure was more effective after local thermal treatment by electrocautery, and protein exudation from lung tissues increased following thermal treatment, which prevented alveolar surface rupture and air leakage. (5) Compared with the existing thermal ablation procedure, TVRS is characterized by a simple protocol, low cost and extensive range of applications.

The major limitations of our study were as follows: (1) The data of the current study were from a single-arm retrospective cohort, and no control group was included to compare the suitability of other surgical methods in this selected cohort. A previous study (8) indicated that thermal ablation with other instruments was no more beneficial than conventional therapy; therefore, it is necessary to perform a prospective, controlled trial to confirm the application range of TVRS. (2) No evidence was found to confirm whether the TVRS procedure can be expanded beyond the patients included in our study to a wider group of patients with severe emphysema, although, in light of its surgical principle, TVRS has some potential benefits.

## Conclusion

In conclusion, we reported a new thermal-based surgical treatment successfully performed in a specific patient group diagnosed with primary lung cancer accompany with pulmonary bullae to reduce the enlargement lung tissue and the procedure did not interfere with the resection of malignant lung tumors. We suggest that thermal-based surgical treatment may be regarded as a potential choice for the treatment of pulmonary bullae in specific clinical situation.

## Data Availability Statement

The raw data supporting the conclusions of this article will be made available by the authors, without undue reservation.

## Ethics Statement

The studies involving human participants were reviewed and approved by the Institutional Review Board of Jilin Cancer Hospital (No. 201412-028). Written informed consent for participation was not required for this study in accordance with the national legislation and the institutional requirements.

## Author Contributions

TL completed the manuscript writing. WL completed the reviewing of manuscript and supplement of image and video. All authors completed study design, data collection, and analysis.

## Conflict of Interest

The authors declare that the research was conducted in the absence of any commercial or financial relationships that could be construed as a potential conflict of interest.

## Supplementary Material

Supplementary Video 1 The burning procedure in TVRS, and the application of using gauze soaked in normal saline can be applied to cover the operating surface during the burning procedure.
